# Editorial: Physiological aspects of marathon running, volume II

**DOI:** 10.3389/fphys.2026.1991611

**Published:** 2026-09-25

**Authors:** Yoshio Suzuki, Daichi Sumi, Henrique Pereira Neiva

**Affiliations:** 1 Graduate School of Health and Sports Science, Juntendo University, Chiba, Japan; 2 Institute of Sport Science, ASICS Corporation, Kobe, Japan; 3 Department of Sport Sciences, University of Beira Interior, Covilhã, Portugal; 4 The Research Center in Sports Sciences, Health Sciences and Human Development (CIDESD), Covilhã, Portugal

**Keywords:** marathon running, pacing strategy, physiological stress, ultramarathon, wearable technology

## 1 Introduction

When we (DS & YS) initiated the first volume of this Research Topic, our vision extended beyond simply curating cutting-edge research articles. We also aimed to attract high-quality review articles that provide broad physiological insights and academic depth, such as Newsholme’s *Keep on Running* (Newsholme et al., 1994).

Starting with this second volume, the editorial team was expanded with the inclusion of another editor (HPN), which contributed to the broader disciplinary scope of the collection. This enhanced collaboration has facilitated the remarkable growth of the collection, which has expanded from seven foundational articles in Volume I to 19 diverse contributions in Volume II. This collection has developed into a cross-disciplinary forum, bridging the gap between basic physiological principles and elite athletic practice, effectively fulfilling the original vision for this Research Topic series. In the following sections, we summarize these contributing works within their respective thematic categories.

## 2 Training systems and determinants of performance

In the first volume (Volume I), the primary focus was on analyzing performance determinants in specific contexts, such as cross-country running (Rapp et al., 2025), and general reviews of pacing strategies (Grivas, 2025). Volume II significantly expands upon these foundational insights, shifting toward the comparative analysis of “training systems” practiced by world-class elite runners, as well as highly specific performance prediction models and individual case reports.

A highlight of this volume is the conceptual comparison between East African and Norwegian endurance training systems. Grivas et al. provide a detailed analysis of two distinct yet successful pathways to elite performance: the “group-based, high-variability training” (e.g., Fartlek) characteristic of Kenyan and Ethiopian runners, and the “lactate-guided threshold density model” that has gained global attention through the success of Norwegian athletes. Their work suggests that elite physiological adaptation is not limited to a single model but can be achieved through different organizational approaches to training intensity and density.

Furthermore, advancements in practical performance prediction have moved beyond the general indicators seen in Volume I. Oficial-Casado et al. proposed a high-precision prediction equation for marathon finish times using a large-scale dataset (over 8,000 participants) from the Valencia Marathon, demonstrating that finish times can be predicted with 85% accuracy based solely on half-marathon performance and sex. There is also a deepening focus on specific runner populations; a case report by Zając et al. documented the preparation of two middle-aged women aiming for a sub-4-hour finish. This study highlights how a 26-week multifaceted intervention—incorporating training load management, nutritional education, and body composition monitoring—contributed to a significant (approximately 20%) improvement in their performance.

Finally, new insights into the impact of terrain on race strategy have been added to the collection. A longitudinal case study of an elite half-marathoner over four years examined how moderate elevation changes influence pacing regulation (Grivas and Alkasasbeh). The findings suggest that topography is not an isolated factor but interacts complexly with the athlete’s physiological state and the specific race context. Together, these studies reflect a transition in marathon research from theoretical physiological frameworks to a phase that addresses more individualized and practical real-world demands.

## 3 Nutritional strategies and the effects of supplements

In the first volume (Volume I), the nutritional focus was primarily on the mechanisms of gastrointestinal (GI) dysfunction with prolonged running and how prolonged endurance exercise interacts with gastrointestinal function and the gut microbiota (Sumi and Suzuki, 2025), such as how probiotics (*Lactobacillus pentosus* CQZC02) might mitigate intestinal barrier dysfunction (Cai and Wang, 2024). Volume II advances these foundational insights toward practical solutions, presenting rigorous evaluations of innovative energy delivery systems and randomized controlled trials on the biochemical modulation of physiological stress via supplementation.

A key contribution to this volume is the study by Zhang et al., which investigated the impact of sodium alginate energy gels on amateur runners during a real-world marathon. Utilizing continuous glucose monitoring (CGM) devices, they demonstrated that alginate-based hydrogels—which encapsulate carbohydrates in the stomach’s acidic environment for sustained release in the small intestine—significantly enhanced glycemic stability, particularly during the mid-race segments. Notably, this technology was found to improve pacing stability across the entire race for elite runners and during the first half for advanced runners. These findings suggest that the “quality” of carbohydrate delivery directly influences biomechanical consistency, bridging the gap between metabolic regulation and race performance in a competitive setting.

Conversely, the collection also includes work that highlights the limitations of specific supplements against the profound stress of marathon running. Schoenfeld et al. conducted a randomized, double-blind, placebo-controlled subanalysis of the Enzy-MagIC trial to examine whether supplementation with hydrolytic enzymes and flavonoids (Wobenzym^®^) could modulate exercise-induced changes in hemostasis markers. Their results showed no significant differences between the supplement and placebo groups regarding markers of coagulation and fibrinolysis, such as D-dimer and prothrombin fragment 1 + 2. The authors suggested that the physiological strain of a marathon is so substantial that any potential immunomodulatory effects of the supplement may be too minor to be detected under such extreme conditions.

Collectively, these studies address the long-standing challenge of integrating nutrition and training—a central pillar in the work of Newsholme (1994) (Newsholme et al., 1994)—by utilizing advanced delivery technologies and detailed hematological profiling. They provide a modern scientific perspective that redefines the boundaries of effective nutritional intervention in the face of intense athletic demand.

## 4 Wearable technologies and real-time monitoring

In the first volume (Volume I), the primary focus was on the physiological interpretation of mobility data, such as the proposal of the HYPAC system for estimating oxygen consumption using GPS (Hata et al., 2024). Volume II expands the scope of wearable technology applications. It moves beyond basic tracking to visualize complex physiological processes, including the impact of advanced footwear technology (AFT) in competitive settings (Muniz-Pardos et al. 2025; Maruo and Takezawa), real-time monitoring of sweat (Bandiera et al.; Ferrari et al.) and glucose (Ishihara et al.; Zhang et al.) dynamics, and the long-term interaction between training load and sleep patterns (Xu et al.).

A prominent topic in this volume is the impact of Advanced Footwear Technology (AFT) on performance. Maruo and Takezawa analyzed extensive datasets from the World Marathon Majors (WMM), revealing that while AFT significantly improved finish times for both sexes, it also induced more aggressive pacing strategies—particularly among female runners—leading to steeper late-race decelerations. Furthermore, Muniz-Pardos et al. highlighted the substantial inter-individual variability in physiological responses to AFT during outdoor running and provided essential methodological guidelines to enhance the ecological validity of future research.

In the realm of sweat and hydration management, innovative technologies have emerged as alternatives to traditional patch-based methods. Ferrari et al. and Bandiera et al. validated the validity and reliability of novel wearable sensors for continuous monitoring of sweat conductivity and sodium concentration during exercise. Although these devices do not yet match the precision of laboratory gold standards, they are recognized as highly practical “plug-and-play” tools that facilitate real-time, individualized hydration strategies in the field.

Advancements were also made in the integrative interpretation of biological data. Ishihara et al. utilized continuous glucose monitors (CGMs) to propose a three-phase conceptual framework for glucose dynamics during ultramarathons, suggesting that late-stage glucose elevations may reflect impaired muscular uptake linked to cumulative inflammatory stress. Additionally, Wang et al. focused on heart rate–speed decoupling in adolescent runners, demonstrating that this phenomenon serves as a durability-related indicator reflecting thermal strain in early stages and energy supply regulation in later stages. Finally, a year-long monitoring study by Xu et al. elucidated a bidirectional lagged relationship between training load and sleep characteristics in recreational runners, where daily training volume influenced subsequent sleep structure, and sleep deficiency limited the following day’s activity.

## 5 Physiological stress and adaptation: immune, blood, and organ systems

In the first volume (Volume I), research in this domain focused primarily on describing correlations between specific biomarkers and gastrointestinal functions (Sumi and Suzuki, 2025, 2026), such as the impact of myokines like decorin on cardiometabolic health (de Fontes-Junior et al., 2025) and the protection of the intestinal barrier via probiotics (Cai and Wang, 2024). Volume II significantly deepens this perspective, incorporating “systems biology simulations (Tian et al.),” “quantitative comparisons of immune and inflammatory responses across varying race distances (Bizjak et al.),” and “the neural control of exercise limitations (Yamanaka et al.),” thereby elucidating the comprehensive scope of marathon-induced stress from both micro- and macro-perspectives.

In the realm of immunology, this volume presents highly contemporary approaches. Tian et al. utilized an Agent-Based Model (ABM) to simulate changes in immune function following a four-week block of high-intensity training (HIT) in professional athletes. Their model visualized a unique “resolution failure” phenotype, characterized by an inversion of the CD4+/CD8+ ratio and the suppression of the anti-inflammatory cytokine IL-10. Furthermore, Bizjak et al. directly compared different ultramarathon distances (100 km, 160.9 km, and 230 km), revealing that while leukocyte dynamics show acute adaptations regardless of distance, the longest races elicit significantly more pronounced cortisol and C-reactive protein (CRP) responses (Bizjak et al.). Regarding hematological and respiratory adaptations, Volume II adds profound insights into both cellular mechanics and long-term training effects. Grau et al. investigated the effects of a 230-km non-stop ultramarathon on red blood cell (RBC) deformability. They discovered that despite the risk of hemolysis from mechanical stress, a compensatory mechanism—driven by the activation of RBC-nitric oxide synthase (RBC-NOS) and S-nitrosylation of spectrins—functions to maintain or even enhance RBC deformability during extreme exertion. Additionally, Nagorka-Bolek et al. compared the pulmonary function of amateur marathoners with a control group, reporting that habitual endurance training is associated with significantly higher forced vital capacity (FVC) and forced expiratory volume in one second (FEV1).

Finally, the role of the central nervous system in defining the limits of human performance is addressed in a pivotal review. Yamanaka et al. outlined how brain circuits, particularly involving the amygdala, regulate cardiovascular responses during high-intensity exercise. They suggested that the central nucleus of the amygdala (CeA) may act as a “central brake” that senses negative emotional states and extreme stress, modulating sympathetic drive to prevent systemic collapse through allostatic regulation. Collectively, these diverse studies illustrate that the marathon runner’s body does not simply suffer from exhaustion; rather, it deploys multiple physiological adaptation mechanisms ranging from the cellular level to complex neural networks.

## 6 Integration and summary of historical data

In the first volume (Volume I), the summaries were primarily focused on specific physiological niches, such as mini-reviews on gastrointestinal function (Sumi and Suzuki, 2025) or psychophysiological perspectives on negative split pacing (Grivas, 2025). In contrast, Volume II provides a grander synthesis by integrating over a century of competitive history and five decades of scientific data from the Comrades Marathon—the world’s oldest and largest ultra-marathon (Knechtle et al.).

The pivotal narrative review by Knechtle et al. serves as the culmination of this volume, synthesizing 42 specific publications to establish a comprehensive clinical and physiological profile of ultra-distance running. This historical analysis elucidates profound demographic shifts, documenting how the field expanded from an exclusively male domain prior to 1975 into a more professionalized environment where the performance gap between sexes is progressively narrowing. Furthermore, the finding that peak performance age in this 90-km race occurs later than in standard marathons (approximately 29.9 years for men and 36.0 years for women) provides empirical support for the long-term durability and efficiency discussed elsewhere in this volume.

Clinical insights derived from this century-long “natural laboratory” also transcend what is possible through single-race observations. The data reveal an unusually high prevalence of acute kidney injury (AKI) and hypernatremia compared to other ultra-endurance events, highlighting the unique renal and electrolyte stress imposed by prolonged, high-intensity exertion in challenging environments. Crucially, however, the review confirms that the vast majority of these disturbances—including cardiac biomarker elevations and muscle inflammation—are transient and functional in nature, reflecting the sophisticated adaptive capacity of the human body.

Ultimately, the process of consolidating fifty years of evidence into a coherent framework fulfills the original vision for this Research Topic series: to transform isolated physiological observations into systematic knowledge that bridges the gap between basic science and elite athletic practice.

## 7 Conclusion

Volume II further expands the scope of this Research Topic by bringing together 19 contributions addressing multiple aspects of marathon physiology. From the comparative analysis of elite training models to the evaluation of advanced wearable technologies and the exploration of immunometabolic and neural control of physiological stress, these works collectively provide diverse perspectives on physiological aspects of marathon and endurance running.

By integrating modern technologies with the foundational principles established by seminal work of Newsholme (Newsholme et al., 1994), this collection effectively bridges the gap between basic physiological science and elite athletic practice. Building upon this sustained success and the growing interest from the global research community, we are pleased to announce that the launch of Volume III has been officially confirmed. We are confident that this Research Topic series will continue to serve as a vital academic guide and a driving force for further advancements in the field, supporting the dual goals of optimizing performance and maintaining athlete health.
